# Systems Analysis of N-Glycan Processing in Mammalian Cells

**DOI:** 10.1371/journal.pone.0000713

**Published:** 2007-08-08

**Authors:** Patrick Hossler, Bhanu Chandra Mulukutla, Wei-Shou Hu

**Affiliations:** Department of Chemical Engineering and Materials Science, University of Minnesota, Minneapolis, Minnesota, United States of America; RIKEN Genomic Sciences Center, Japan

## Abstract

N-glycosylation plays a key role in the quality of many therapeutic glycoprotein biologics. The biosynthesis reactions of these oligosaccharides are a type of network in which a relatively small number of enzymes give rise to a large number of N-glycans as the reaction intermediates and terminal products. Multiple glycans appear on the glycoprotein molecules and give rise to a heterogeneous product. Controlling the glycan distribution is critical to the quality control of the product. Understanding N-glycan biosynthesis and the etiology of microheterogeneity would provide physiological insights, and facilitate cellular engineering to enhance glycoprotein quality. We developed a mathematical model of glycan biosynthesis in the Golgi and analyzed the various reaction variables on the resulting glycan distribution. The Golgi model was modeled as four compartments in series. The mechanism of protein transport across the Golgi is still controversial. From the viewpoint of their holding time distribution characteristics, the two main hypothesized mechanisms, vesicular transport and Golgi maturation models, resemble four continuous mixing-tanks (4CSTR) and four plug-flow reactors (4PFR) in series, respectively. The two hypotheses were modeled accordingly and compared. The intrinsic reaction kinetics were first evaluated using a batch (or single PFR) reactor. A sufficient holding time is needed to produce terminally-processed glycans. Altering enzyme concentrations has a complex effect on the final glycan distribution, as the changes often affect many reaction steps in the network. Comparison of the glycan profiles predicted by the 4CSTR and 4PFR models points to the 4PFR system as more likely to be the true mechanism. To assess whether glycan heterogeneity can be eliminated in the biosynthesis of biotherapeutics the 4PFR model was further used to assess whether a homogeneous glycan profile can be created through metabolic engineering. We demonstrate by the spatial localization of enzymes to specific compartments all terminally processed N-glycans can be synthesized as homogeneous products with a sufficient holding time in the Golgi compartments. The model developed may serve as a guide to future engineering of glycoproteins.

## Introduction

Since the introduction of tissue plasminogen activator two decades ago, many recombinant proteins produced by mammalian cells have become important therapeutic biologics. A vast majority of these recombinant proteins are glycoproteins. The extent of glycosylation and the structure of the glycans on those glycoproteins have a profound effect on their biological activities and circulatory half-life (for review see [1], [2]). Depending on their attachment site on the polypeptide, these glycans are either O-linked (through serine or threonine) or N-linked (through asparagine on the Asn-X-Thr/Ser recognition sequence). N-glycans in particular have an important role in protein folding in the endoplasmic reticulum (ER). Unlike O-glycosylation, which has been shown to initiate in either the ER or Golgi [3], N-glycosylation is initiated by transfer of a preassembled oligosaccharide (Glc_3_Man_9_GlcNAc_2_) to Asn at the binding site of a nascent protein translocating into the ER lumen [4]. Although all N-glycans are linked to a protein molecule through Asn-X-Thr/Ser, not all of these motifs are occupied by an N-glycan. Hence, there exists different permutations of site occupancies in proteins which frequently have multiple N-glycan binding sites. This phenomenon is referred to as macroheterogeneity. Before exiting the ER, three glucose, and usually at least one mannose sugar are removed from the N-glycan. The removal of glucose serves as a quality control for proper folding of these glycoproteins and their readiness for transit to the Golgi apparatus [5].

Inside the Golgi, more mannose sugars are removed before further extension of the glycan branches. Subsequent step-wise addition of sugars to different positions of the extending glycan is catalyzed by a number of glycosyltransferases, each adds a particular monosaccharide through a specific glycosidic bond. Most intermediate glycans along the biosynthetic pathway in the Golgi have more than one available reaction site, either on the same or different sugar moieties, for receiving a monosaccharide. In some cases the reactions of adding those sugars to the glycan may occur in different sequential orders. In others, the addition of a particular glycosidic linkage hinders the reaction of the others [4]. Along the N-glycan biosynthesis pathway there are usually only a relatively small number of glycosyltransferases each capable of catalyzing different glycosidic linkages on different N-glycans, and are used multiple times along the pathway. This web of reactions forms a complex network which, when drawn out graphically, indeed resembles a network of diverging and converging paths leading to a number of different fully-extended N-glycan structures. The glycans are thus rather diverse. Adding to this diversity, many glycans do not achieve terminal processing, but exit with the protein in the form of intermediately processed glycans.

N-glycan structures are generally classified into three principal categories: high mannose, complex and hybrid types. All of them share a common tri-mannosyl (Man_3_GlcNAc_2_) core structure. The high mannose glycans have 5 to 9 mannose (Man_5–9_GlcNAc_2_) sugars. Those with 2 GlcNAc's attached to the tri-mannosyl core are called complex type. As its name implies, the hybrid type are a combination of high mannose and complex glycans, and have at least three mannose sugars, but only one GlcNAc on one nonreducing mannose. This diversity of glycan sugar composition on each glycosylation site is referred to as microheterogeneity.

N-glycan microheterogeneity thus arises through enzyme processing in the Golgi apparatus. The Golgi apparatus is consisted of stacks of membranous compartments commonly grouped into cis, medial, trans, and TGN (trans-Golgi network) cisternae. These cisternae are not biochemically homogeneous. As the secretory glycoproteins traverse through these Golgi compartments the glycan extension reactions are catalyzed by varying compositions of glycosylation enzymes in each compartment. There are still unresolved questions on the nature of Golgi compartmentalization and different models on intra-Golgi transport have been proposed [6], [7]. In the vesicular transport model the different stacks of cisternae (or compartments) are seen as stationary while the secretory cargo is transported by vesicles which bud off one stack and fuse to the next. In contrast, the Golgi maturation model envisions the secretory cargo as being relatively stationary with respect to the stack or compartment in which they reside; while each compartment undergoes a maturation process to transform from early cisternae to late cisternae. Although recent visualization studies on yeast seem to favor the maturation model [8], [9], the controversy persists [10].

From a classical reaction engineering perspective the two models present two different idealized reactor scenarios. The vesicular transport model resembles a model of continuous stirred tank reactors in series (CSTR's). The glycoprotein molecules enter the first compartment and after a finite amount of holding time they are continuously transported to the next one downstream. Conversely, protein processing in the Golgi maturation model is better represented by a non-stationary batch reactor. The reactor thus closely resembles a plug-flow reactor (PFR), the traversal of protein over time in the maturation model can be viewed in the same way as traveling downstream in a tubular reactor. A key distinction between the two models is that in the maturation model all protein molecules entering the Golgi together will exit at the same time, while in the vesicular transport model, mixing in each compartment facilitates different protein molecules residing in each compartment for different lengths of time. Common to both models is that the enzyme composition varies as the glycans proceed downstream. Since the time each protein molecule spends in the Golgi compartments affects the extent of glycan extension, the distribution of residence times will have a profound effect on their N-glycan structure.

The microheterogeneity of N-glycans on glycoproteins can thus be attributed to the nature of the glycosylation reactions and the mixing characteristics of the Golgi apparatus as proteins traverse the system. With the clinical importance of glycoprotein therapeutics, there is a strong desire to better control the variability of glycoforms of those products. A better understanding of kinetics of the reactions involved and the maturation process of Golgi apparatus will certainly aid in the control of microheterogeneity of glycoprotein products. However, with the reaction kinetic parameters difficult to determine experimentally, and with the model of Golgi processing still being debated, experimental assessment of the source of microheterogeneity and exploration of their control is difficult. An alternative to experimental investigation is through mathematical modeling to assess the effectiveness of metabolic intervention towards the channeling of glycans under different models.

Previous studies have applied mathematical models to evaluate protein N-glycosylation microheterogeneity [11], [12]. Those two studies modeled the Golgi as four continuous well-mixed reactors in series. In light of recent experimental evidence that favors the maturation model [8], [13], we evaluated the differences between different reactor characteristics on the resulting glycan distribution. This work presents the simulation results through a visualization tool we have previously reported [14] to allow for quick assessment of the effects of various parameters (see Table S1 in the Supporting Information) on the glycan flux distribution.

## Materials and Methods

### System Description

The model of N-glycosylation considers the core N-glycan biosynthetic pathway in the Golgi apparatus common to most host cells used for the expression of recombinant proteins. The pathway consists of a large number of reactions catalyzed by a relatively small number of enzymes. The ability of many of these enzymes to catalyze the same reaction on different glycan substrates, and the fact that most glycans can possibly be a substrate of more than one enzyme, makes this pathway form a highly branched, initially divergent, and eventually convergent network. Since the model considers glycosylation reactions in the Golgi, the input into the system is considered to be a 9-mannose glycan on a single Asn-x-Thr/Ser glycosylation site that can be processed in the Golgi.

**Figure 1:**
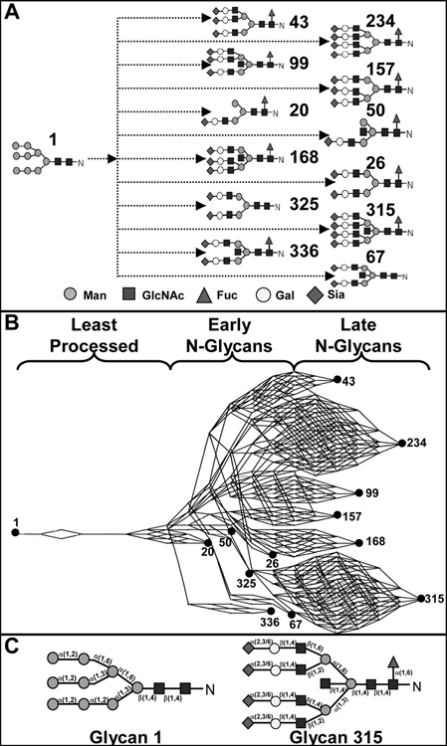
Glycan reaction network and its symbolic representation. A: Schematic of the formation of the terminally processed N-glycans; dashed lines denote multiple reaction steps. B: The corresponding network representing the various pathways connecting the glycans shown in A; least, early, late, and terminally processed glycans denoted on the network, as plotted by GlycoVis. C: The starting glycan and the most fully processed N-glycan considered in the model, with sugar linkages labeled for reference.

The N-glycosylation enzymes considered in this model are shown in Table 1. The substrate specificities and their glycan intermediates and products have been described previously [14]. There exist a large number of pathways connecting the respective glycans towards the initial 9 mannose glycan that we consider to be the root of the biosynthetic tree (Fig. 1A). Figure 1B shows a pathway representation of this initial N-glycan (glycan 1) entering the pathway with that of the twelve fully processed N-glycans considered in this model, and discussed more extensively in the subsequent sections. Least processed N-glycans are considered to be the non-terminally processed, high mannose, hybrid, and incompletely processed biantennary glycans, early N-glycans include terminally processed biantennary glycans as well as non-fully galactosylated tri- and tetraantennary glycans, and late N-glycans are considered to be the more fully galactosylated tri- and tetraantennary glycans. Figure 1C highlights the glycosidic linkages of some of these glycans to serve as a reference.

**Table 1:** List of enzymes considered with their reported and calculated parameter values.

Enzyme (e_m_)	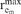 (µM/min/cell)	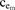 (µM)	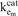 (min^−1^)	Reference	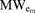	Reference
Man I	611	19.9	30.1	[17]	230,000	[18]
Man II	5797	6.6	883.7	[18]	285,000	[19]
GnT I	1358	1.7	790	[20]	46,500	[21]
GnT II	671	0.5	1314	[22]	43,000	[23]
GnT III	12	0.2	56	[24]	114,000	[25]
GnT IV	29	0.3	94	[26]	63,200	[26]
GnT V	2637	2.2	1188	[27]	69,000	[27]
FucT	2969	15.4	192	[28]	44,000	[29]
GalT	1235	80.2	15	[30]	440,000	[31]
SiaT	2328	1.6	1502	[32]	40,500	[33]

**Figure 2:**
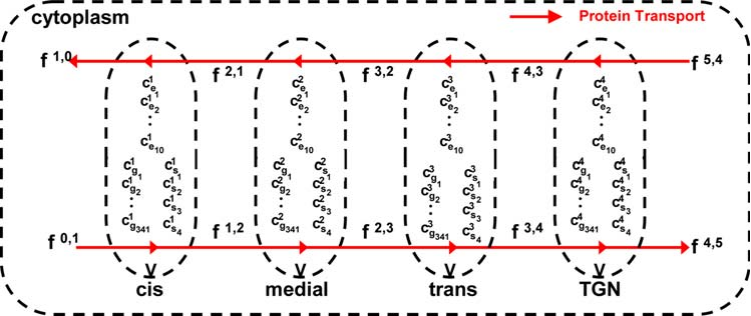
Schematic of the Golgi as a system of four reactors in series.

The model considers glycosylation reactions occurring in four compartments of equal volume in series, representing the cis, medial, trans, and trans-Golgi network (TGN) compartments. Inter-compartmental transport of glycosylated protein molecules is limited to adjacent compartments via retrograde and anterrograde transport. The glycoprotein is transferred from the ER into the first compartment. From each subsequent compartment the glycans are transferred to the next, eventually being secreted from the last compartment in an anterrograde manner. The volume of each compartment is assumed to be constant; implicitly balanced by anterrograde transport from the upstream compartment. For simplification, no retrograde transport or the reverse flow of the glycoprotein is considered. The holding time for glycoproteins in each compartment is set to be the same. A schematic of the Golgi system is shown in Figure 2.

The enzyme composition in each compartment may differ as reported [15]. The mixing behavior of the enzymes and glycans involved in the glycoconjugate reactions in each compartment affects the glycan profile. The enzymes within each compartment are considered to be uniformly distributed and their concentrations at steady state. The distribution of the N-glycans (and the protein molecules they are conjugated to) in each compartment is modeled as that of either well-mixed reactor (CSTR), or a plug flow reactor (PFR) as discussed in the Introduction section. It is assumed that anterrograde transport is non-selective, thus in the CSTR model all glycoproteins in a particular compartment can be transported. In contrast, in the PFR model, only those glycoproteins that have reached the distal end of the compartment are transported. In this context, the well-mixed model is a representation of the vesicular transport model in which the glycoprotein molecules traversing through the four compartments have a distribution of residence times which is dictated by the mixing characteristics of the Golgi. Whereas the PFR model represents the maturation model, in which all glycoprotein molecules are confined to the same physical entity until secretion.

Mass balances on the N-glycan species for each of the compartments in the CSTR model give the following:
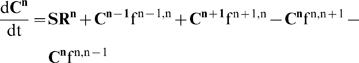
(Eq.1) Where superscript n denotes the compartment n, C is the concentration vector for the N-glycans, and S is the stoichiometric matrix. R is the reaction rate vector dependent on both kinetic constant values, and the concentration of glycan and nucleotide-sugar substrates. The last four terms on the right hand side of Eq. 1 describes the anterrograde and retrograde transport of glycans between compartments n, n-1, and n+1. In the CSTR model all glycoprotein molecules are transported at a constant rate f. The balance of these transport terms keeps the volume of each compartment constant.

In the PFR model both space and time are independent variables as shown below,
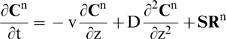
(Eq.2)where v is the linear velocity. In the case of the Golgi maturation model, all the protein molecules are held in the same cisternae for N-glycan processing, and the dispersion of the molecules is assumed to be negligible. The transport between adjacent compartments is described by boundary conditions at the entrance (z = 0) and exit (z = l) of the compartment. At z = 0,
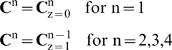
where 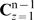 are the vector of glycan concentrations at the exit of the previous compartment, and 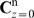 are the vector of glycan concentrations entering the system. At steady state, when the concentration profiles of all glycans are well-established in the reactor, the equation becomes
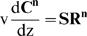
(Eq.3)


For both the CSTR and PFR models the anterrograde transport from the ER into the cis compartment is assumed to include only the initial 9-mannose glycan attached to the glycoprotein as input into the entire system. Unless specified, it is assumed that the amount of glycoproteins contributed from retrograde transport in the other compartments is negligible.

### Enzyme Kinetics

Among the ten enzymes considered, two (Man I and Man II) exoglycosidase enzymes use glycans as their sole substrate, while the other eight (GnT I-V, FucT, GalT, SiaT) glycosyltransferases use both N-glycans and the corresponding nucleotide-sugar as substrates. The one substrate enzymes were considered to follow Michaelis-Menten kinetics with substrate competition. The two substrate enzymes were assumed to follow a rapid equilibrium, random, bi-bi mechanism. Each of the glycosyltransferases can add a monosaccharide to multiple glycans through the formation of a specific glycosidic bond. Those glycans are treated as competing substrates. For the one substrate enzymes, the equation for the p^th^ reaction catalyzed by enzyme e_m_ in compartment n is expressed as:
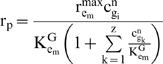
(Eq.4)Similarly, for the two substrate enzymes:
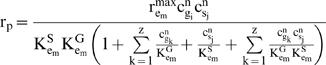
(Eq.5)In both equations, g_i_ is any glycan which can be a substrate of e_m_, and in the case of the latter, s_j _refers to the corresponding nucleotide-sugar (see Text S1 in the Supporting Information). The two summation terms in the denominator of Eq. 5 take into consideration the competitive inhibition of N-glycan substrates towards the enzymes, which reduce the individual reaction rates. The enzymes that react upon the N-glycans towards the terminal stages of processing (GalT, SiaT) have the largest number of potential substrates (Table 2). As a result, the denominator term is quite large for these enzymes.

**Table 2:** List of 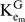
and 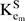 values used in the model.

Enzyme (e_m_)	# of Glycan Substrates	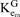 (µM)	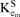 (µM)	Reference
Man I	8	17	N/A	[17]
Man II	6	200	N/A	[35]
GnT I	1	260	170	[36]
GnT II	7	190	960	[22]
GnT III	7	21	420	[24]
GnT IV	13	800	220	[26]
GnT V	12	87	11000	[27]
FucT	11	25	46	[28]
GalT	232	660	22	[30]
SiaT	234	67	28	[37]

### Parameter Values

The concentrations and 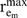 values of the enzymes involved in glycan biosynthesis vary widely among different cell lines. The 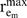 values and enzyme concentrations that have been reported in the literature for the enzymes considered are listed in Table 1 [16]-[32]. These values were estimated from maximum reaction rates and enzyme concentrations reported for each enzyme in the literature, assuming a total cellular protein content of 250×10^−9^ mg per cell (see Text S2,S3 in the Supporting Information). It was further assumed that the volume of each Golgi compartment (V) was equal to 10^-13^ ml, as estimated using electron microscopy of Golgi cisternae [33]. In the cases that multiple glycan reactants are substrates for the same enzyme, the 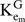 values were assumed to be equal for each glycan (Table 2) [16], [21], [23], [25]-[27], [29], [34]-[36].

Unless specified otherwise, 186 molecules/s of glycoprotein was used as the rate of input from the ER into the Golgi. This number was chosen as it corresponds to a productivity of 40 pg/cell/day of protein (MW 150 kDa), a level commonly seen in highly secretory cells, and possibly closer to the levels in the types of cells reported in the literature [37] from which the enzyme concentrations and kinetic constants were used. Each molecule is assumed to have one glycosylation site. The mean residence time of a protein molecule (as well as the glycan) in each compartment is assumed to be 10 min. The vesicular transport rate is 10^−14^ ml/min unless specified otherwise.

The ten enzymes were considered to have a spatial localization across the Golgi cisternae. The mannosidases are generally localized towards the cis/medial regions of the Golgi, the N-acetylglucosaminyltransferases and fucosyltransferases are localized towards the medial region, and the galactosyltransferases and sialyltransferases are localized towards the trans and trans-Golgi network (TGN) regions of the Golgi. In our model, unless otherwise specified, Man I-II are assumed to be localized in the four compartments in a [0.15, 0.40, 0.30, 0.15] ratio; GnT I-V were localized in a [0.20, 0.45, 0.20, 0.15] ratio; and GalT and SiaT in a [0, 0.05, 0.20, 0.75] ratio [38]. The intra-Golgi concentration of nucleotide-sugars has been reported to be in the mM range, at levels, 10–50 fold higher than that in the cytosol [39], [40]. Unless specified otherwise, the concentration of each individual nucleotide-sugar is assumed to be the same in all of the compartments.

### Model Simulation and Parameter Estimation

**Figure 3:**
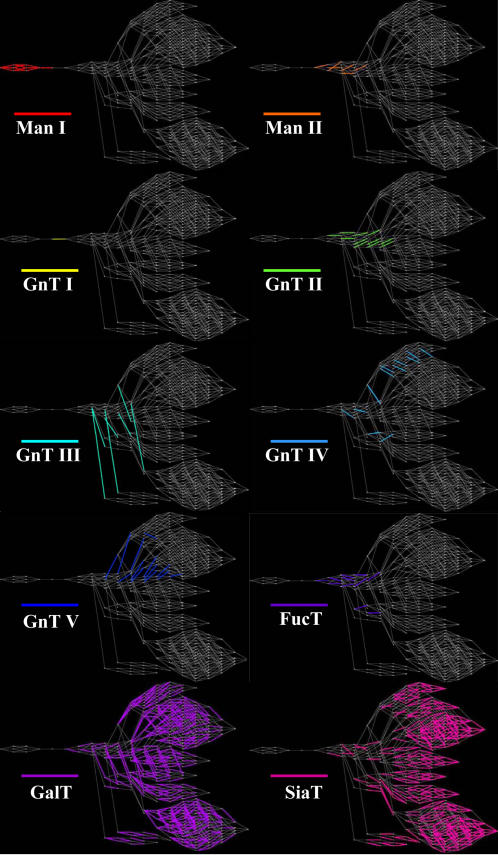
Pathway maps with reactions highlighted according to the enzyme catalyzing the reaction.

The model equations were solved in Matlab (Mathworks, Inc.) using an implicit ODE solver. The initial glycan concentrations were all assumed to be zero, except the first initial 9 mannose glycan, whose concentration was set equal to the total protein concentration. All calculated solutions were stable steady states with negative, real eigenvalues. The simulation results are displayed through a pathway visualization program, GlycoVis [14]. Representative pathway maps for the system are shown in Figure 3, where the reactions catalyzed by each of the 10 enzymes are highlighted using a different color.

An objective of this study was to gain insight on the effects of different variables on the resulting glycan distribution. We sought to obtain a set of parameter values for which all the terminally processed glycans are well represented in the product mixture, so that any perturbation introduced can be better seen. While keeping the kinetic parameters constant (i.e., 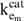, 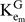, and 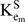 values), we identified a set of enzyme and nucleotide-sugar concentrations that allow for a relatively even distribution of the twelve terminally processed N-glycans.

The method of steepest descent in a Matlab program was used to adjust the enzyme and nucleotide-sugar concentrations that give rise to a mixture of terminal glycans of a specified pattern. Specifically, in the initial evaluation of the model the enzyme and nucleotide-sugar concentrations that produce the terminal glycans in the same order of magnitude were used as listed in Table 3. Later in the study the method was applied to search conditions that will produce a single terminal N-glycan. The objective function that was minimized is shown in Eq. 6, where 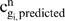 is the concentration of glycan species g_i_ in compartment n predicted at each iteration of the solver, and 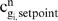 is the desired value.
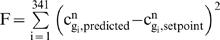
(Eq.6)The enzyme and nucleotide-sugar concentrations obtained were values of a local minimum of the objective function, not a global minimum. Nevertheless, these values enable us to better discern the changes of various fluxes under different simulation conditions.

**Table 3:** Enzyme and nucleotide-sugar concentrations calculated through nonlinear optimization.

Enzyme (e_m_)	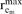 (µM/min/cell)	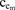 (µM)
Man I	920	29.7
Man II	1220	1.4
GnT I	950	1.2
GnT II	1893	1.4
GnT III	165	2.9
GnT IV	809	8.6
GnT V	1020	0.9
FucT	322	1.7
GalT	345	23
SiaT	710	0.5

### Sensitivity Analysis

The degree to which an enzyme controls metabolic pathway fluxes can be represented by sensitivity coefficients for fluxes,
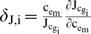
(Eq.7)where 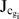 is the flux of the N-glycan exiting the Golgi. Positive sensitivity coefficients indicate a direct relationship between changes in enzyme levels with that of the exiting fluxes. Negative coefficients indicate an inverse relationship. Sensitivity analysis can also be taken with respect to other variables such as nucleotide-sugar concentrations.

## Results

### Glycan Processing in a PFR Model

#### Effect of processing time

**Figure 4:**
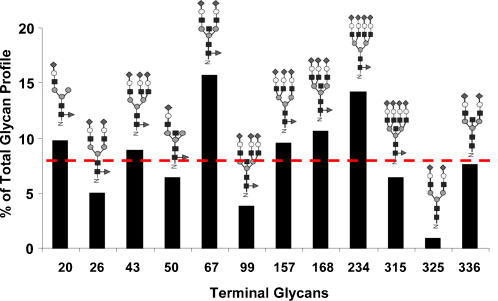
Percentage of the terminally processed N-glycans after adjusting the enzyme levels in a single PFR model (dashed line corresponds to a completely equal mixture).

The model we have constructed is a generic one with most enzymes commonly seen in rodent and human cells in culture. However, the relative abundance levels of these enzymes vary amongst different cell lines. Instead of focusing on simulations for a particular cell line, we employ the model to illustrate key features of the glycosylation system that gives rise to glycan microheterogeneity and affects the glycoprotein quality. With the parameter values used, a holding time of 40 minutes is sufficient to allow all glycans to reach the terminal glycans in every branch. To probe the kinetic behavior of the glycosylation system, we examined the system operating like an ideal plug flow reactor with a normalized distance from entry of 1, (i.e., time divided by 40 minutes) sufficiently long for a complete conversion of all glycans to the terminally processed glycans. The initial 9-mannose glycan that enters the compartment reacts to form intermediate glycans as it moves downstream. At different time points after its entry (corresponding to a fixed position downstream from the entry point along the Golgi system), a snapshot of the concentration profile of all glycans, and the flux of every reaction was taken. The enzyme and nucleotide-sugar concentrations used were obtained from nonlinear optimization, as explained in the Model section, and are listed in Table 3 for the results shown in Figure 4. They were further assumed to be uniform throughout the compartment in order to better discern the effects of residence time. The reaction rates at different points in the compartment are affected only by the glycan.

**Figure 5:**
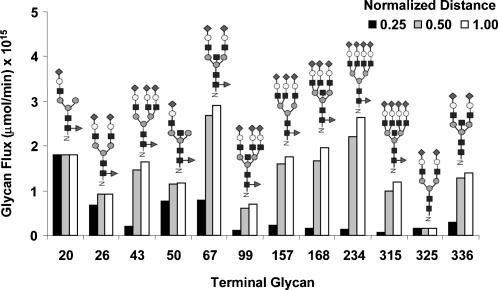
Effect of normalized distance (holding time) on glycan flux in a PFR model.

**Figure 6:**
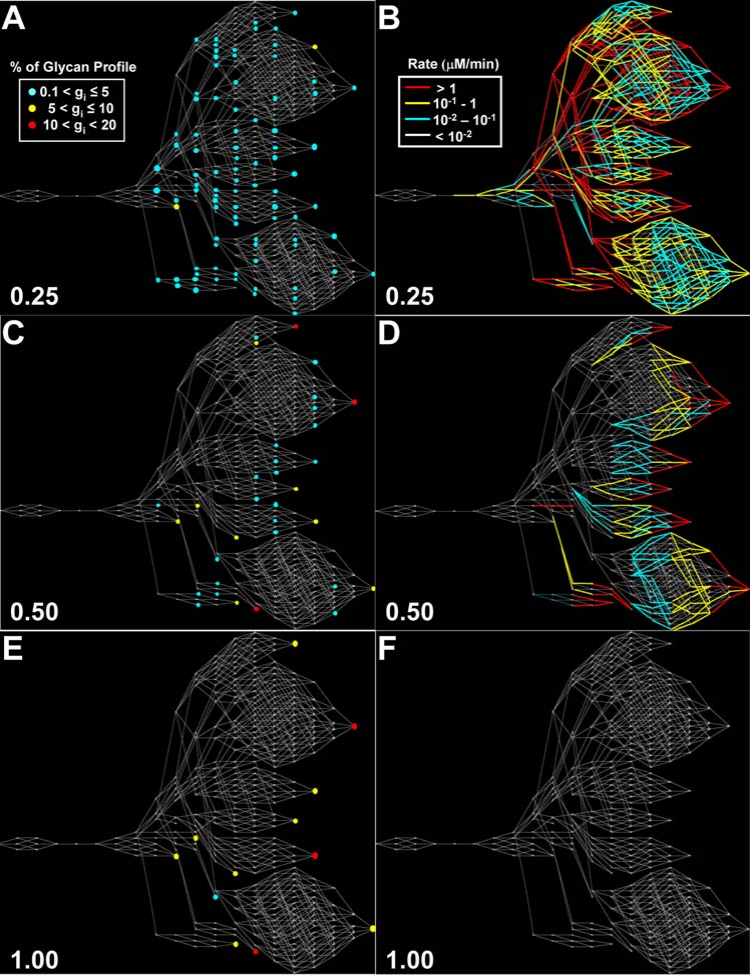
Visualization of the glycan profile and relative reaction rates as a function of normalized distance. A,B: 0.25 C,D: 0.50 E,F: 1.0

The twelve terminally processed N-glycans at different time points after the initial glycan enters the Golgi are shown in Figure 5. As time progresses the concentration of most terminally processed glycans increase. After 40 min of processing all of the initial glycan input has reached the terminal glycans. Using the visualization program GlycoVis, the concentrations of every glycan in the network, as well as the flux of each particular reaction at 10, 20, and 40 minutes were visualized as shown in Figure 6. As the glycans move downstream in the Golgi, more complex glycans begin to appear. But at any given position a heterogeneous mixture of glycans is present. Given sufficient time all glycans can reach terminal processing and the number of glycans converges to twelve. As described in materials and method the enzyme concentration needed in the simulation was adjusted to give a flux profile of all terminal glycans in the same order of magnitude. The flux distribution plot (Fig. 6B,D,F) demonstrates that the reactions involved in final glycan extension are less active in the front end of the Golgi (Fig. 6B), while the early glycan processing reactions are less active in the distal end of the Golgi (Fig. 6F). However, many enzymes catalyze multiple reactions in the network and are utilized more uniformly throughout, catalyzing reactions involving different glycan substrates in different parts of the Golgi.

#### Effect of pathway branching on glycan microheterogeneity

In a reaction system with branched pathways, like the protein N-glycosylation pathway, the branch points determine the distribution of fluxes and are the controlling points where fluxes can be directed towards a particular product. However, the glycosylation reaction system is a complex system with an extensive substrate spectra for many of the enzymes involved. Except for GnT I, which reacts upon only one glycan, and Man I which reacts upon the earliest high mannose glycans, the other enzymes are involved in multiple branches of the pathway. In fact, these eight enzymes generated 326 glycans in the pathway. Many enzymes involved in a reaction at a particular branch point are also involved in other downstream reactions in the same, and/or different branches. Channeling the flux at a branch point by amplifying the enzyme involved, or by suppressing another enzyme catalyzing a competing reaction, will invariably cause changes in other downstream reactions. The end results thus may not be as expected.

**Figure 7:**
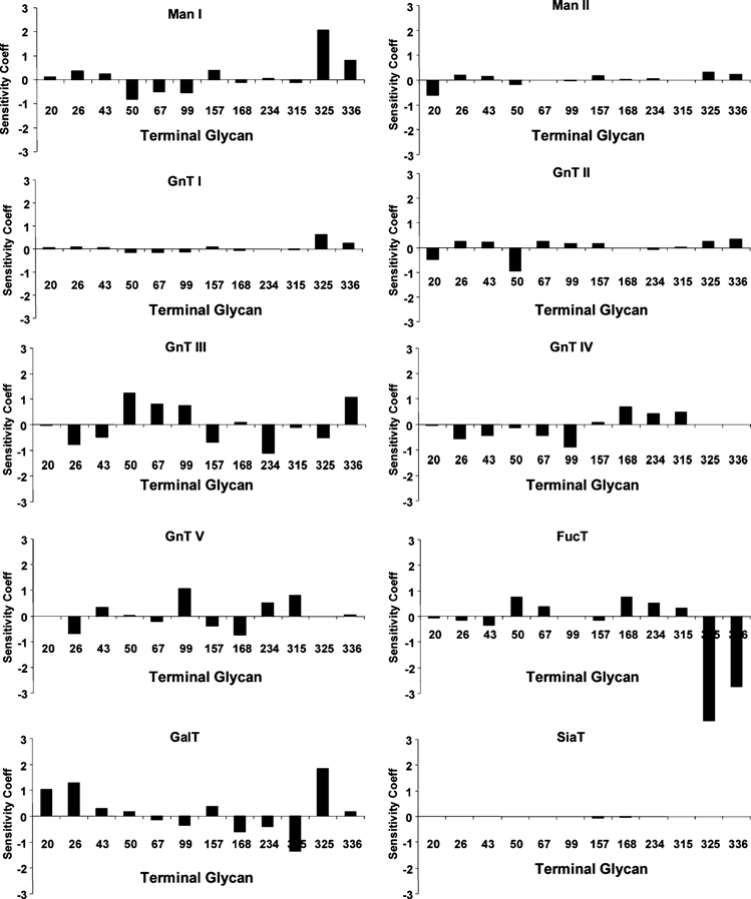
Sensitivity coefficients of the 12 terminal glycans with respect to the ten enzymes.

To assess the effect of different enzyme levels on the resulting N-glycan profile, sensitivity coefficients of the 12 terminally processed glycans were calculated with respect to the 10 enzymes after 40 minutes; sufficiently long for all reactions to complete (Fig. 7). As expected, changing the level of any particular enzyme, except SiaT, had a rather profound effect on the terminal glycan flux. Most enzymes had a strong effect on multiple branches in the pathway. It is noted that even Man I and Man II, the two enzymes catalyzing the initial reactions prior to the glycan extension reactions, also had a rather profound influence on terminal glycan composition. Upon closer examination, this is attributed to the effect of their reaction rate on the downstream concentrations of glycans at branch points. Altering the glycan concentration at the branching nodes has an effect on the ratio of fluxes diverging from the branch point because of differences in 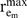
and 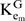 values of these different enzymes for the same glycan substrate. The fact that no single, terminally processed glycan has a prevailing positive sensitivity coefficient suggests that upon changing the level of an individual enzyme, more than one glycan will be affected.

**Figure 8:**
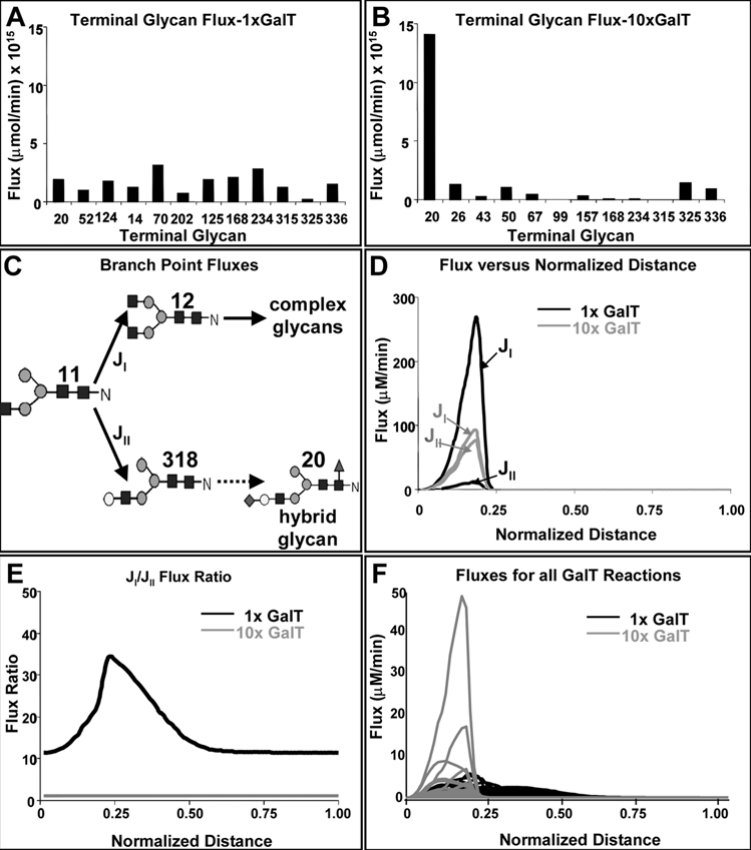
The effect of GalT amplification on N-glycan fluxes. A: Terminal glycan fluxes with basecase GalT concentration. B: Terminal glycan fluxes with 10X higher GalT concentration. C: Controlling branch point in N-glycan biosynthesis. D: Comparison of fluxes at branch point shown in C, at different GalT concentrations, and normalized distances. E: Ratio of fluxes at the branch point, at different GalT concentrations. F: Fluxes of all GalT catalyzed reactions, at different GalT concentrations and normalized distances.

Figure 8 illustrates an example of the effect of altering GalT which gives a high sensitivity coefficient for the flux of glycan 20. One glycan substrate of GalT, glycan 11, is positioned at a pivotal branch point of the network. Besides accepting Gal to its β(1,2) GlcNAc to form glycan 318 (as shown in Fig. 8C), it can also add GlcNAc to its α(1,6) Man through GnT II forming glycan 12. However, the addition of galactose to glycan 11 lowers the selectivity of the glycan for GnT II, thus committing the glycan towards the formation of hybrid type glycans [4]. This branch point thus affects the ratio of complex and hybrid type glycans on the glycoprotein. Increasing the level of GalT increases the flux towards glycan 318, and downstream glycan 20. The simulation results of the glycoform distribution after 40 minutes are compared between the base case GalT enzyme concentration with that of a 10-fold increase in GalT concentration (Fig. 8A,B). The fluxes around this branching point are also shown (Fig. 8C,D). As shown in Figure 8D, their rates change as the glycoproteins move downstream in the compartment. To better illustrate those fluxes around this pivotal point, their dynamics along the Golgi is shown. The fluxes for all of the reactions are initially low and gradually rise. This is expected near the beginning since the substrates have not yet been formed. However, after reaching a high activity region it sustains for some duration before subsiding again. Note that the relative magnitude of the fluxes catalyzed by GalT and GnT II differ quite a bit upon increasing GalT by 10-fold. Nevertheless, the flux to complex glycan 12 is not zero. It is readily seen how the ratio of fluxes towards glycans 12 and 318 decreased as a result of this perturbation (Fig. 8E). It should be kept in mind that an important variable not considered in this one compartment model is enzyme localization. One would expect that localization would have an important role towards ensuring that glycan 20, and other less commonly observed hybrid type glycans, are not measured in abundance.

The results shown in Figure 8 demonstrate that increasing GalT by ten-fold does channel most of the flux towards hybrid type glycan 318, and away from complex type glycan 12. However, GalT reacts with 231 other N-glycans as can be seen in Figure 3 in addition to glycan 11. Increasing GalT concentration affects all those reactions. As a result, higher flux is not only observed in J_1 _but also in other GalT catalyzed reactions (Fig. 8F). In addition, as can be seen from the kinetic expression in Eq. 5, even though the concentration of those competing substrates may be low, their combined effect may be profound on the resulting galactosylation of glycan 11.

#### Channeling glycans to reduce microheterogeneity

**Figure 9:**
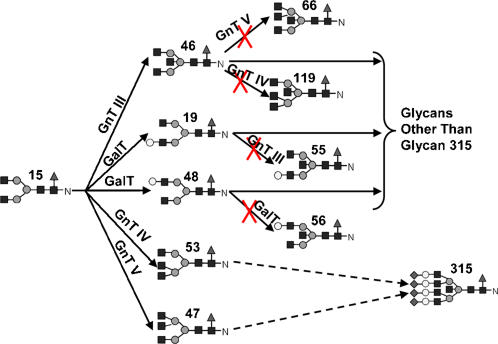
Competing reactions utilizing glycan 15.

To evaluate the feasibility of redirecting the fluxes to near structural uniformity by metabolic engineering, nonlinear optimization was applied with enzyme levels as adjustable variables. Using the nonlinear optimization algorithm we explored the possibility of altering enzyme concentrations to direct the fluxes to a single terminal glycan. The abundance level attainable for each one of the 12 terminal N-glycans, and the percentage of the other terminal glycans along with the levels of each enzyme are shown as a column in Table 4. For some terminal glycans, a very high level of uniformity (glycans 26, 325 and 336) can be achieved. However, for others (glycans 99, 315), a high abundance level is not attainable. A particular example worth noting is channeling fluxes to glycan 315, a fully sialylated, and tetraantennary glycan. Its percentage of the total N-glycan profile did not exceed 85% even upon major changes in enzyme levels. As illustrated in Figure 9, at a key branch point, GnT III, GnT IV, GnT V, and GalT all compete for the same substrate glycan 15. GnT III, GnT IV and GnT V add an N-acetylglucosamine to glycan 15 to three different positions. Ostensibly their reactions may occur in different orders, but will eventually give rise to the same glycan, although through different intermediates; and even lead to the same final glycan 315. However, once GnT III reacts upon glycan 15 (giving rise to glycan 46), GnT IV and GnT V have a lower specificity towards it; and once GalT reacts upon glycan 15 (transforming it to glycans 19 or 48), GnT III has a lower specificity toward it. GalT and GnT III reactions on glycan 15 thus lead to the diversion of glycan extension reactions away from glycan 315. To maximize glycan 315 formation, GnT IV and GnT V enzyme levels need to be sufficiently high to compete against GalT and GnT III, whose levels must be comparatively smaller, as seen in Table 4. Ideally one would eliminate GnT III and GalT to block the two branches diverting glycan 15. However, those two enzymes are required in downstream reactions along the branches catalyzed by GnT IV and GnT V to lead to glycan 315. Eliminating those diverting branches will thus also abrogate the synthesis of glycan 315. Therefore, manipulation of enzyme levels alone will not direct most of the fluxes to glycan 315. Another alternative would be to control the order at which these enzymes react upon glycan 15.

**Table 4:** Enzyme concentrations sufficient to achieve a high percentage of each terminal N-glycan.

Enzyme (e_m_)	Enyzme Concentration^a^ 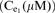												
Man I	(29.7)	11.4	183.8	53.4	384.0	139.7	16.7	74.8	318.8	34.6	40.7	271.0	168.9
Man II	(1.4)	2.5	9.3	6.9	6.8	7.1	6.6	6.8	8.4	6.6	5.4	39.6	12.7
GnT I	(1.2)	0.6	3.6	1.3	1.7	2.1	1.4	6.3	6.3	2.2	8.7	5.4	4.1
GnT II	(1.4)	2.9	7.1	5.4	4.6	6.8	5.3	5.7	6.5	0.7	38.0	38.1	38.1
GnT III	(2.9)	0.0	0.0	0.0	332.1	16.8	3.3	0.0	14.2	0.0	9.8	0.0	94.3
GnT IV	(8.6)	77.2	0.0	0.0	7.8	0.0	0.0	49.6	92.8	32.5	119.6	1.6	1.6
GnT V	(0.9)	1.3	0.0	8.2	2.2	0.0	4.0	0.0	0.0	3.4	8.9	0.1	0.1
FucT	(1.7)	29.7	16.0	15.6	17.7	13.6	15.1	15.8	18.8	16.9	8.8	0.0	0.0
GalT	(23)	2535.6	9.7	7.5	5.6	8.4	5.7	10.6	7.1	8.6	13.7	8.6	8.0
SiaT	(0.5)	1.6	1.5	1.5	1.6	1.6	1.5	1.5	1.3	6.4	1.4	1.3	1.3

Glycan (g_i_)	Glycan % of Glycoform Profile^a^												
g_20_	(9.7)	96.3	0.7	1	0.3	0.7	1.1	1.2	0.9	3.7	0.8	0	0
g_26_	(5.0)	2	99.3	1.2	0	4.4	1.7	2.4	0.7	0	0	0	0
g_43_	(8.9)	0.1	0	97.8	0	0	17.1	0	0	1.1	0.9	0	0
g_50_	(6.3)	0	0	0	94.3	13.1	8.2	0	7.3	0	1.9	0	0
g_67_	(15.7)	0	0	0	2.9	81.4	18.7	0	9.6	0	12.4	0	0
g_99_	(3.7)	0	0	0	0	0	53.3	0	0	0	7.1	0	0
g_157_	(9.5)	1.6	0	0	0	0	0	96.3	5.9	1.0	0.6	0	0
g_168_	(10.6)	0	0	0	0.1	0	0	0	75.3	0	8.9	0	0
g_234_	(14.2)	0.1	0	0	0	0	0	0	0	94.2	12	0	0
g_315_	(6.4)	0	0	0	0	0	0	0	0	0	53.8	0	0
g_325_	(0.8)	0	0	0	0	0	0	0	0	0	0	100	0.1
g_336_	(7.5)	0	0	0	2.5	0.4	0	0	0.4	0	1.7	0	99.9

a Parentheses denote the basecase simulation values for the enzyme concentrations, and glycan %'s.

#### Effect of compartmentalization and enzyme spatial localization

**Figure 10:**
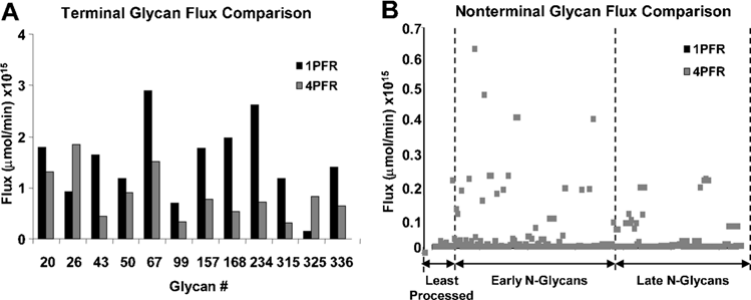
Comparison of N-glycan fluxes between 1PFR and 4PFR models. A: Terminal glycan fluxes. B: Nonterminal glycan fluxes.

The simulations shown above are based on uniform flow and assume the Golgi apparatus behaves as a single compartment. With the PFR assumption whether the Golgi is treated as a single compartment or multiple compartments in series the reaction characteristics are the same. However, with different enzyme distributions in the four compartments, the reaction kinetics will be different from those of single compartment. To assess the effect of a spatial distribution of enzymes, a simulation was performed using the same set of parameters as those used for the results shown in Figure 4. Each of the four PFR has a normalized distance of 0.25 or a holding time of ten minutes. The distribution of enzymes in the four compartments was described in the Parameter Values section. Within each compartment the enzymes are assumed to be uniformly distributed. For comparison, the simulation results of the 1PFR and 4PFR models are both shown in Figure 10. The most significant effect of spatial distribution of enzymes is a decrease in flux for most of the terminal glycans and the emergence of non-terminal glycans (Fig. 10A,B respectively). The representation of upstream enzymes is reduced in later Golgi compartments. As a result, the early glycans which are relatively “under-processed” in the early compartment will be even more likely to remain unprocessed and appear in the final product.

### Glycan Processing In A Well-mixed, Compartmentalized Model

The simulations performed with varying residence times in the plug flow model demonstrated that an insufficient holding time can lead to incomplete processing. The glycoform profiles observed experimentally often include those from early (high mannose type), to intermediate, and terminally processed glycans [41]. A possible mechanism leading to the emergence of early glycans in the secreted product is through a distribution of residence times of glycans caused by mixing in the compartments of the Golgi. As envisioned in the vesicular transport model, the glycoprotein cargo in a particular compartment is enclosed in transport vesicles and transferred to the next compartment downstream. The 4CSTR model assumes that the protein molecules in each compartment are well-mixed and all molecules have equal probability of being engulfed in the transport vesicle, which is certainly an idealized case. The transport between compartments is only downstream, that is, the retrograde transport was again assumed to be negligible. Only glycans in the last compartment (TGN) are allowed to be secreted from the system as final products. The parameters used were the basecase values shown in Tables 2 and 3, with the same enzyme localization as the 4PFR simulations.

**Figure 11:**
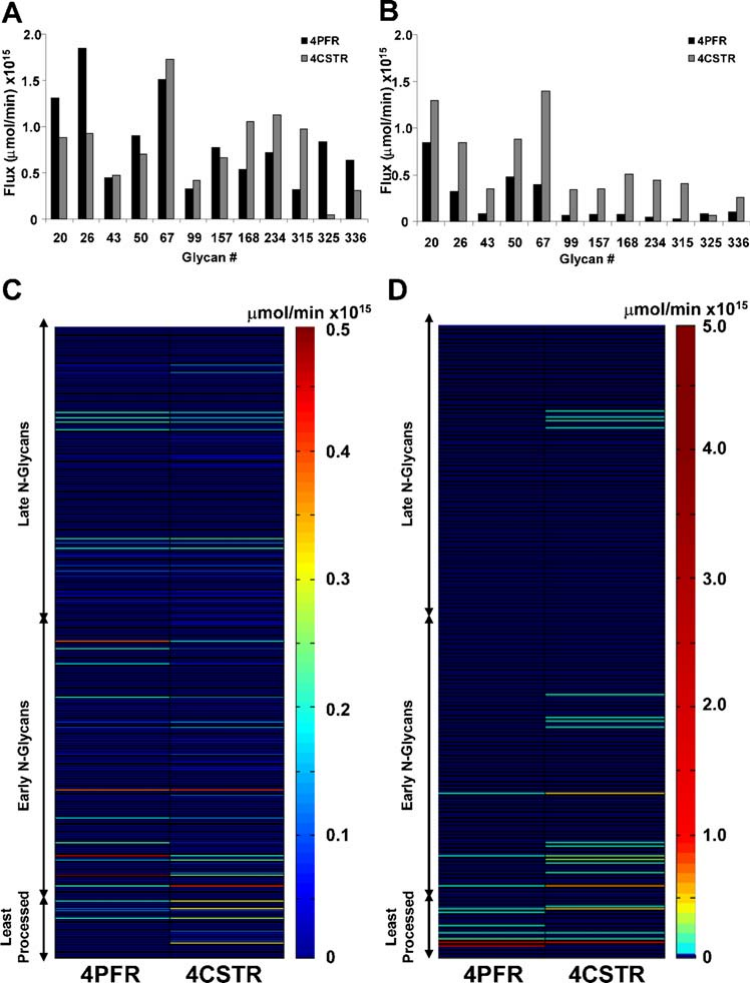
Comparison of N-glycan fluxes between 4PFR and 4CSTR models. A: Terminal glycan fluxes with a 40 min residence time. B: Terminal glycan fluxes with a 20 min residence time. C: Nonterminal glycan fluxes with a 40 min residence time; least, early, and late processed glycans as shown in Figure 1B. D: Nonterminal glycan fluxes with a 20 min residence time; least, early, and late processed glycans as shown in Figure 1B.

Of the 12 terminally processed glycans, some have a shorter reaction path leading to their formation (“early” terminal N-glycans) whereas others have a longer reaction path (“late” terminal N-glycans). The positions of these two different terminal glycan types on the reaction network are highlighted in Figure 1B. The steady-state terminal glycan distribution in the secreted product evaluated using the 4CSTR model is shown in Figure 11A, and compared to the results from the 4PFR model with a 40 minute holding time. The 4CSTR model showed a decrease in the early terminal glycans, including glycan 20 and an increase in the late terminal glycans. The secretion rates for the non-terminally processed N-glycan products obtained in the 4PFR and 4CSTR systems are shown as heat maps in Figure 11C. The rate of secretion of each glycan is depicted by the color of the corresponding line segment. With the long holding time that was used, the glycoform profile change of terminal and non-terminal glycans are not very profound with the parameter values used for simulation. It thus appears that the effect of changing from a 4PFR to a 4CSTR system is much less dramatic than the case of changing from the 1PFR to the 4PFR model with a spatial enzyme distribution (Fig. 10). However, if indeed each compartment resembles a CSTR, glycans will have a wide distribution of holding times when exiting to the next compartment. It will certainly result in an increased heterogeneity in the last compartment and thus in the secreted product. With a sufficiently long holding time the non-terminal glycans constitute only a small fraction of total glycans. In the case that the holding time is shorter, an increased heterogeneity, with a higher presence of intermediate glycans is certainly expected. We examined the case in which the residence time of glycans was set at 20 minutes (5 minutes in each compartment) and the results are shown in Figures 11B and 11D. In this case the contrast between 4PFR and 4CSTR is even more vivid. As expected, the fluxes of the terminal glycans are reduced from those attained at the 40 minute residence time, but the decrease is more profound with 4PFR than with 4CSTR (Fig. 11B). These glycans in the 4PFR system appear as high mannose glycans because of insufficient reaction time in the early compartments. The glycans which do not get processed in the early compartment will not get extended in the downstream compartments if the required enzymes are not sufficiently localized in the downstream compartments. A closer examination of the composition of the non-terminal glycans reveals that except for the very least processed N-glycans, the early and late intermediate N-glycans are present at higher levels in the 4CSTR system, and are more heterogeneous (Fig. 11D). This is expected in a mixing reactor system.

### Channeling Of Fluxes to Produce Homogeneous Glycoform Profiles

The 4PFR model simulation results points to how high mannose glycans can become a rather significant fraction of the product glycans as has been reported [41]. A contributing factor towards this phenomenon is through enzyme localization; the responsible enzymes are present in abundance only in the early compartment, the unreacted glycans thus remain unprocessed and are secreted with the other more fully processed N-glycans. The 4PFR model does predict a high proportion of very early glycans. The 4PFR model also simultaneously predicts the presence of a smaller number of intermediate glycans at a significant level; whereas the 4CSTR model favors a wider spectrum of intermediate glycans. The reported glycoform profiles tend to show a number of significantly abundant glycans, rather than having a larger number of insignificantly abundant glycans.

To assess whether glycans on a glycoprotein can be directed to become “uniform” we applied the non-linear optimization algorithm to vary the spatial distribution of enzymes. The objective function was set to channel the fluxes in the 4PFR model towards glycan 99, a triantennary glycan with a bisecting N-acetylglucosamine. In the 1PFR model this glycan could not be produced as a homogeneous product through the manipulation of enzyme levels alone (Table 4). We thus chose this glycan to illustrate that through spatial enzyme localization, it can be secreted theoretically as a uniform product. The enzymes levels in each compartment that facilitate the formation of uniform glycan 99 are listed in Table 5. Not every enzyme is present in every compartment. Due to the glycan substrate distribution in each compartment only a fraction of the possible reactions for each enzyme have an active flux in each compartment. For example comparing GnT V in the medial cisternae and all the reactions it catalyses (Fig. 3), only a small fraction is active. Similar applications of the nonlinear optimization algorithm towards varying the spatial enzyme distribution for achieving uniformity of the other terminal N-glycans was also successful. Hence, both enzyme concentration and localization are important towards the redistribution of fluxes through the N-glycan biosynthetic pathway.

**Table 5:** Enzyme concentrations and their relative distribution and category in each compartment required to achieve a high proportion of terminal N-glycan 99.

Enzyme (e_m_)	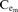 (µM)	Enzyme Fold^a^			
		cis	medial	trans	TGN
Man I	29.7	0.8 (I)	0 (II)	0 (II)	0 (II)
Man II	1.4	0 (II)	0.8 (I)	0 (II)	0 (II)
GnT I	1.2	0 (II)	0.8 (I)	0 (II)	0 (II)
GnT II	1.4	0 (II)	0.8 (I)	0 (II)	0 (II)
GnT III	2.9	0 (II)	0 (III)	1.6 (I)	0 (II)
GnT IV	8.6	0 (II)	0 (III)	0 (III)	0 (II)
GnT V	0.9	0 (II)	2.4 (I)	0 (II)	0 (II)
FucT	1.7	0 (II)	1.5 (I)	0 (II)	0 (II)
GalT	23	0 (II)	0 (III)	0 (III)	3.0 (I)
SiaT	0.5	0 (II)	0 (II)	0 (II)	0.8 (I)

Terminal Glycan (g_i_)	Flux (µmol/min)×10^15^	% of Glycans
20	0	0
26	0	0
43	0	0
50	0	0
67	0	0
99	18.51	100
157	0	0
168	0	0
234	0	0
315	0	0
325	0	0
336	0	0

a Parentheses denote the category of each enzyme in each of the compartments.

**Figure 12:**
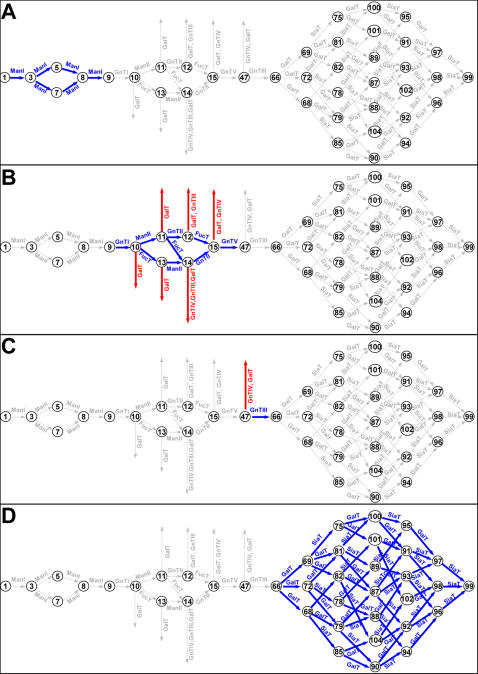
Active and divergent reactions in each compartment for the generation of uniform glycan 99. A: Cis Golgi, B: Medial Golgi, C: Trans Golgi, D: TGN Golgi. Arrows: blue for reactions with active flux; gray for inactive fluxes; red for divergent fluxes.

The 10 enzymes considered can be classified into three categories for each compartment. Category I enzymes are those which are essential for channeling glycans to the homogenous end product, category II enzymes are those whose presence in the compartment is neither essential nor detrimental, category III enzymes are detrimental and cause divergence of fluxes and preclude the formation of glycan homogeneity. A number of enzymes in particular compartments are category II; their concentrations in those compartments can be from zero to very high without any consequence on the glycan flux profile. For clarity, the concentrations of category II enzymes in those particular compartments are kept at zero. The entries in Table 5 are labeled depending upon the category of the enzyme in each compartment. A pictorial representation for the distribution of enzymes in the four compartments is also presented in Figure 12 with enzymes in categories I, II and III, shown in blue, grey, and red background respectively. Those reactions which are active (have positive flux) are depicted by thicker, colored edges, corresponding to the type of enzyme responsible for the reaction.

Although only the results for glycan 99 are shown, all the other terminal N-glycans could be made to a uniform product given a sufficient holding time and enzyme localization (data not shown). Controlling the order of monosaccharide addition onto the N-glycan, while simultaneously taking advantage of enzyme substrate specificities, facilitates the formation of a less heterogeneous glycoform profile. Experimentally to accomplish the channeling of fluxes towards a homogeneous product may entail localizing category I enzymes in the designated compartments, while simultaneously suppressing category III enzymes in the same compartment. In the case of glycan 99, enzymes Man I, Man II, GnT I, GnT II, GnT V, FucT, SiaT fall into either category I or category II for different compartments. GnT III is a category III enzyme for the medial but falls into category I for the trans compartment. Similarly GalT is a category III enzyme for both the medial and trans compartments, but is an essential enzyme for the final compartment. In the case of GnT IV, even though it doesn't participate in the synthesis of glycan 99, its presence in the medial or trans compartments increases the heterogeneity in the end product, and diverts fluxes away from the glycan.

## Discussion

Microheterogeneity of N-glycans is ubiquitously seen in mammalian cell derived recombinant glycoproteins. Since glycan microheterogeneity is a natural phenomenon seen even in glycoproteins in the body, its occurrence per does not pose a concern for therapeutic proteins. However, different glycoforms of therapeutic proteins may affect the pharmacokinetic behavior and the activity of the protein therapeutic. As a result, controlling the microheterogeneity to be within a defined quality range, or even directing the glycan synthesis to the most effective forms is highly desirable. Most glycan intermediates involved in the reaction system can serve as a substrate for multiple enzymes. The ten enzymes considered unequally share 329 glycan substrates. Such extensive competition for these glycan substrates by different enzymes gives the reaction network the look of an intricate web with multiple branches. Rationally engineering the pathway to channel the fluxes towards a preferred glycan may not be an easy task without the aid of careful analysis. In this study we examined a number of factors contributing to heterogeneous glycan distribution.

We analyzed the glycosylation reactions at two levels. First the reactions were simulated in a single plug flow reactor to examine the intrinsic reaction kinetics; then we proceeded to examine their behavior in a simulated Golgi apparatus, considering it to be 4PFR in series or 4CSTR in series. The two models resemble the two hypotheses on cargo movement across the Golgi; the cisternal maturation and vesicular transport models.

The reaction paths and reaction time to form fully processed N-glycans makes the residence time of the glycoprotein in the Golgi apparatus an important variable. Given a sufficiently long reaction time, all glycan reactants in the input will be converted to fully processed glycans, as can be seen in Figure 5. To reduce the complexity we chose to use a long residence time for most analyses presented. This residence time is within the range of values reported in the Golgi [42]. Furthermore, to observe the flux distribution of all branches involved, the parameter values were adjusted from those obtained from literature to allow for fluxes of the twelve fully processed glycans fall within the same order of magnitude. The highly interactive nature of the network makes the rates of reactions highly dependent on one another. Not only do the reactions in the same branch, or in the local region of network affect each other, but also those in different branches or at different distances in the network (Fig. 3). No single enzyme plays a dominating role in channeling the flux to a particular terminal glycan as illustrated by the distributive nature of the sensitivity coefficients (Fig. 7). Changing the levels of most enzymes, except SiaT, affects the flux at multiple branches. The difficulty of channeling glycan fluxes by simply manipulating a single enzyme level is illustrated by the example of amplifying GalT as shown in Figure 8. To effectively channel the glycans one may have to resort to changing the levels of multiple enzymes depending on the target glycan. As shown in Table 4, effective channeling towards relative glycan abundance can be accomplished with many of the terminal N-glycans, but not all.

Since the Golgi is compartmentalized, and is generally thought as having multiple cisternae, it is prudent to examine the glycosylation reactions as such. The Golgi maturation model envisions the Golgi compartments as maturing from early to late cisternae, with its internal content (including enzymes) evolving throughout the maturation process. This time evolution can be viewed as spatial progress over time, just like a plug-flow reactor. We modeled this system as four tubular reactors in series allowing the enzyme concentration to vary in each compartment. An obvious effect of extending from 1PFR to 4PFR is the presence of non-terminal N-glycans in the final product. The holding time used for simulation was more than sufficient for completion of reaction to terminal glycans in a single PFR, nevertheless a substantial amount of non-terminal glycans are seen. The disappearance of early enzymes as the Golgi becomes mature prevents some intermediately processed glycans from achieving terminal processing.

A deviation from plug flow can potentially increase the extent of microheterogeneity since different glycoprotein molecules stay in each compartment for different lengths of time. Indeed, the phenomenon of residence time distribution of secreted protein molecules from the Golgi has been reported [42]. The vesicular transport model is described as 4 CSTR's in series. In other words, the content in each compartment is well-mixed, and the content that the vesicles carry is identical to the compartment they bud off from. The time that a protein molecule spends inside the ER, Golgi, and in the secretory pathway before being secreted varies with the nature of the molecule, ranging from 28 minutes for α_1_-protease inhibitor to over two hours for transferrin [43]. Furthermore, for a given protein not all of its molecules spend an equal amount of time traversing through those organelles. Rather, a wide distribution of residence time is seen. This certainly points to a mechanism of secretory transport that is different from an ideal plug flow model.

By modeling the Golgi apparatus as 4 CSTR's in series the heterogeneity in intermediately processed glycans does increase at a shorter residence time as compared to 4PFR in series. (Fig. 11) More species of non-terminal glycans are present if the compartments are treated as a CSTR. As expected, with a very long holding (or reaction) time, more glycans are terminally processed, and the degree of heterogeneity is less.

The simulation of glycan distribution is by no means sufficient to support one model over the other. The true model is likely neither perfect PFR nor perfect CSTR, but a composite between them. Indeed, this has been hypothesized previously [6], [44]. Nevertheless, taken together the simulation results appear to favor the maturation model which simulates the process as four tubular flow reactors in series. As seen in Figure 11D, with a shorter holding time, the difference between the two models is very profound. Many early glycans appear in the secreted glycoprotein products in both the 4CSTR and 4PFR models. In the 4CSTR system the emergence of early glycans is accompanied by many other glycans. However, in the 4PFR model the early glycans can possibly appear as more significant quantities due to enzyme localization. The emergence of those early glycans resembles experimental observation with both intracellular and secreted N-glycans. Indeed, previous studies have shown an abundance of intracellular high mannose glycans [14], [45].

Although the 4PFR model predicts a glycan distribution that more closely mimics experimental observation, there are other possible mechanisms which may contribute to the degree of N-glycan microheterogeneity. For example a sorting mechanism may selectively allow cargo to move to the next compartment; thus creating different holding times for different glycan species. The mixing of the enzymes within the compartments may not be best depicted as either perfectly well-mixed or plug flow. The Golgi glycosylation enzymes are all type II transmembrane proteins consisting of an amino terminal cytoplasmic tail, a signal anchor transmembrane domain, a stem region, and a large luminal catalytic domain. Although the proteins being glycosylated are in the lumen of the Golgi, the reactions being catalyzed are on the membranes. As protein substrates travel towards and away from the glycosylation enzymes on the membrane, some degree of micromixing is inevitable.

Another key assumption which may have a major effect on microheterogeneity is the relative specificity of the enzymes toward their various substrates. In our model the affinities are assumed to be the same for all glycan substrates for each enzyme. Previous reports have shown that these binding constants are different in some cases [46]. The fact that reported glycans are not as heterogeneous as the above simulations may indicate that substrate affinity may have a prominent role. Unfortunately experimental data on substrate specificity is extremely difficult to obtain, and the literature information is incomplete. With sufficient glycan data one may deduce the relative affinity of different glycans towards the same enzymes.

In this report we notably did not address the issue of the effect of productivity. Of course, the effect of protein secretion rate on its glycan microheterogeneity is of paramount interest for bioprocessing. One should be reminded that a very large fraction of cellular proteins traverse through the ER and Golgi, in addition to the recombinant protein. The glycosylation machinery, at any given moment, is not only processing glycans on recombinant protein molecules, but also all the other cellular proteins that pass through the Golgi. Strictly speaking, in the kinetic equations an expression of other proteins and their associated glycans ought to be included as competing substrates. The mechanism of high specific productivity is not well-understood. Whether the increased productivity is accompanied by a shorter holding time in the Golgi while maintaining the same Golgi volume, or by an increase in Golgi volume while maintaining the holding time, is not clear. Without a better understanding of the mechanism of increased specific productivity, one cannot rationally predict its effect on the glycoform distribution.

The order at which the enzymes react upon the N-glycan helps determine the preferential channeling of the biosynthetic pathways towards the product glycans. The results shown in Figure 12 and Table 5 certainly illustrate the potential benefit of spatial localization in engineering glycoforms and producing a uniform N-glycan product. In recent years there have been numerous reports on the effect of glycans on the activities of therapeutic proteins and on the attempts to engineer the glycan toward the preferred glycoform.[35], [47]–[49]. There has also been an increased interest in producing glycoproteins of “uniform” glycan composition, especially by production in yeast [50]. The interest is spurred not only by the possibility of more comprehensively examining such molecules' biological activities, but also by its potential applications in better formulating biotherapeutic proteins. The mechanism of localization of glycosylation enzymes to different Golgi compartments is still not well-understood. In the vesicular transport model the enzymes are effectively retained in different relative abundances within the cisternae while the cargo is transported through vesicles (for review [51]). Whereas in the Golgi maturation model, the cargo stays in the cisternae as it matures through the secretory pathway. The protein structure requisite for functional retention within the respective cisternae has been studied, and has been found to differ between different protein glycosylation enzymes [52]. Engineering enzyme localization has been successfully used in *Pichia pastoris* towards the generation of uniform, and humanized glycoform profiles [50], [53]. Changing the localization of glycosylation enzyme GnT III has been attempted in mammalian cells [54], which resulted in an increase in the proportion of GlcNAc bisected, non-fucosylated, hybrid-type glycans. This work certainly points to the feasibility of engineering the glycosylation pathway through enzyme localization.

It should also be noted that a large number of proteins transit through the Golgi in addition to the recombinant protein product. Drastically altering the glycosylation pathway may also alter the glycan structure or protein quality of other cellular proteins. Channeling N-glycans to a uniform glycoform thus requires not only information on protein localization, but also a better understanding of the effect of glycan modification on cellular homeostasis. For further exploration of cellular engineering and modulating glycan microheterogeneity one should explore the engineering of enzymatic substrate specificity to direct the N-glycans toward desirable pathways in addition to altering their localization. The model developed in this study can be used to further the exploration of an optimal way of modulating enzymatic substrate specificity in order to alleviate and/or improve N-glycan microheterogeneity towards a desired glycoform profile. Such a systematic approach will continue to enhance our capability in glycoengineering.

## Supporting Information

Table S1 List of variables and their symbolic representation in the model.
(0.07 MB DOC)

Text S1 Reaction rate derivation.
(0.10 MB DOC)

Text S2 Enzyme concentration determination.
(0.05 MB DOC)

Text S3 Maximum reaction rate determination.
(0.05 MB DOC)
