# Supplementary material for: Systems Analysis of N-Glycan Processing in Mammalian Cells
Source: PLoS One. 2007 Aug 8;2(8):e713. doi: 10.1371/journal.pone.0000713 (PMC1933599; doi:10.1371/journal.pone.0000713)
Supplement: Table S1 — List of variables and their symbolic representation in the model. (0.07 MB DOC) [file pone.0000713.s001.doc]

| **Symbol** | **Explanation** |
| --- | --- |
| g_i_ | Glycan i |
| s_j_ | Nucleotide-sugar j |
| e_m_ | Enzyme m |
| **C^n^** | Vector of glycan concentrations in compartment n |
| $\text{c}_{\text{g}_{\text{i}}}^{\text{n}}$ | Concentration of glycan i in compartment n |
| $\text{c}_{\text{s}_{\text{j}}}^{\text{n}}$ | Concentration of nucleotide sugar j in compartment n |
| $\text{c}_{\text{e}_{\text{m}}}^{\text{n}}$ | Concentration of enzyme m in compartment n |
| n | Golgi compartments (1=cis, 2=medial, 3=trans, 4=TGN) |
| f^n,n+1^ | Volumetric flow rate between compartments n and n+1 |
| V | Golgi compartment volume |
| **R^n^** | Reaction rate matrix |
| r_p_ | Reaction rate for the p^th^ reaction |
| $\text{r}_{\text{e}_{\text{m}}}^{\text{max}}$ | Maximum reaction rate of enzyme m |
| $\tilde{\text{r}}_{\text{e}_{\text{m}}}^{\text{max}}$ | Maximum specific activity of enzyme m |
| $\text{k}_{\text{e}_{\text{m}}}^{\text{cat}}$ | Reaction rate constant (turnover number) of enzyme m |
| $\text{K}_{\text{e}_{\text{m}}}^{\text{S}}$ | Dissociation constant of nucleotide-sugar from enzyme m |
| $\text{K}_{\text{e}_{\text{m}}}^{\text{G}}$ | Dissociation constant of glycans from enzyme m |
| $\text{MW}_{\text{e}_{\text{m}}}$ | Molecular weight of enzyme m |
| $\text{PR}_{\text{e}_{\text{m}}}$ | Purification ratio of enzyme m |
| TCP | Total cellular protein |
| Man I | Mannosyl-oligosaccharide 1,2-α-mannosidase (e_1_) |
| Man II | Mannosyl-oligosaccharide 1,3-1,6-α-mannosidase (e_2_) |
| GnT I | α-1,3-mannosyl-glycoprotein 2-β-N-acetylglucosaminyltransferase (e_3_) |
| GnT II | α-1,6-mannosyl-glycoprotein 2-β-N-acetylglucosaminyltransferase (e_4_) |
| GnT III | β-1,4-mannosyl-glycoprotein 4-β-N-acetylglucosaminyltransferase (e_5_) |
| GnT IV | α-1,3-mannosyl-glycoprotein 4-β-N-acetylglucosaminyltransferase (e_6_) |
| GnT V | α-1,6-mannosyl-glycoprotein 4-β-N-acetylglucosaminyltransferase (e_7_) |
| FucT | Glycoprotein 6-α-L-fucosyltransferase (e_8_) |
| GalT | β-N-acetylglucosaminyl glycopeptide β-1,4-galactosyltransferase (e_9_) |
| SiaT | β-galactoside α-2,3/6-sialyltransferase (e_10_) |
| GDP-Fuc | Guanidine diphosphate-fucose (s_1_) |
| UDP-GlcNAc | Uridine diphosphate-N-acetylglucosamine (s_2_) |
| UDP-Gal | Uridine diphosphate-galactose (s_3_) |
| CMP-Sia | Cytosine monophosphate-N-acetylneuraminic acid (s_4_) |

**Table S1**

List of variables and their symbolic representation in the model.
