# Supplementary material for: Systems Analysis of N-Glycan Processing in Mammalian Cells
Source: PLoS One. 2007 Aug 8;2(8):e713. doi: 10.1371/journal.pone.0000713 (PMC1933599; doi:10.1371/journal.pone.0000713)
Supplement: Text S1 — Reaction rate derivation. (0.10 MB DOC) [file pone.0000713.s002.doc]

**Text S1-Reaction Rate Derivation**

The following set of reaction equations were used to derive the rate expressions for the two substrate enzymes:

$$\text{S1.1}$$

Solving simultaneously the following system of equations,

$$\text{S1.2}$$

$$\text{r}_{\text{p}}\text{= k}_{\text{e}_{\text{m}}}^{\text{cat}}\text{c}_{\text{e}_{\text{m}}\text{g}_{1}\text{s}_{\text{j}}}$$

$$\text{c}_{\text{e}_{\text{m}}^{\text{total}}}\text{= c}_{\text{e}_{\text{m}}}\text{+c}_{\text{e}_{\text{m}}\text{g}_{1}}\text{+c}_{\text{e}_{\text{m}}\text{g}_{2}}\text{+c}_{\text{e}_{\text{m}}\text{s}_{\text{j}}}\text{+c}_{\text{e}_{\text{m}}\text{g}_{1}\text{s}_{\text{j}}}\text{+c}_{\text{e}_{\text{m}}\text{g}_{2}\text{s}_{\text{j}}}$$

$$\text{S1.3}$$

$$\text{K}_{\text{e}_{\text{m}}}^{\text{S}}\text{=}\frac{\text{c}_{\text{e}_{\text{m}}}\text{c}_{\text{s}_{\text{j}}}}{\text{c}_{\text{e}_{\text{m}}\text{s}_{\text{j}}}}\text{=}\frac{\text{c}_{\text{e}_{\text{m}}\text{g}_{1}}\text{c}_{\text{s}_{\text{j}}}}{\text{c}_{\text{e}_{\text{m}}\text{g}_{1}\text{s}_{\text{j}}}}\text{=}\frac{\text{c}_{\text{e}_{\text{m}}\text{g}_{2}}\text{c}_{\text{s}_{\text{j}}}}{\text{c}_{\text{e}_{\text{m}}\text{g}_{2}\text{s}_{\text{j}}}}$$

$$\text{S1.4}$$

$$\text{K}_{\text{e}_{\text{m}}}^{\text{G}}\text{=}\frac{\text{c}_{\text{e}_{\text{m}}}\text{c}_{\text{g}_{1}}}{\text{c}_{\text{e}_{\text{m}}\text{g}_{1}}}\text{=}\frac{\text{c}_{\text{e}_{\text{m}}}\text{c}_{\text{g}_{2}}}{\text{c}_{\text{e}_{\text{m}}\text{g}_{2}}}\text{=}\frac{\text{c}_{\text{e}_{\text{m}}\text{s}_{\text{j}}}\text{c}_{\text{g}_{1}}}{\text{c}_{\text{e}_{\text{m}}\text{g}_{1}\text{s}_{\text{j}}}}\text{=}\frac{\text{c}_{\text{e}_{\text{m}}\text{s}_{\text{j}}}\text{c}_{\text{g}_{2}}}{\text{c}_{\text{e}_{\text{m}}\text{g}_{2}\text{s}_{\text{j}}}}$$

$$\text{S1.5}$$

$$\text{r}_{\text{e}_{\text{m}}}^{\text{max}}\text{=k}_{\text{e}_{\text{m}}}^{\text{cat}}\text{c}_{\text{e}_{\text{m}}^{\text{total}}}$$

$$\text{S1.6}$$

results in the reaction rate equation for enzyme m sharing 2 glycan substrates in compartment n:

$$\text{S1.7}$$

$$\text{r}_{\text{p}}\text{ =}\frac{\text{r}_{\text{e}_{\text{m}}}^{\text{max}}\text{c}_{\text{g}_{\text{i}}}^{\text{n}}\text{c}_{\text{s}_{\text{j}}}^{\text{n}}}{\text{K}_{\text{e}_{\text{m}}}^{\text{S}}\text{K}_{\text{e}_{\text{m}}}^{\text{G}}\left(\text{1+}\sum_{\text{k=1}}^{2} \frac{\text{c}_{\text{g}_{\text{k}}}^{\text{n}}}{\text{K}_{\text{e}_{\text{m}}}^{\text{G}}}\text{+}\frac{\text{c}_{\text{s}_{\text{j}}}^{\text{n}}}{\text{K}_{\text{e}_{\text{m}}}^{\text{S}}}\text{+}\sum_{\text{k=1}}^{2} \frac{\text{c}_{\text{g}_{\text{k}}}^{\text{n}}\text{c}_{\text{s}_{\text{j}}}^{\text{n}}}{\text{K}_{\text{e}_{\text{m}}}^{\text{G}}\text{K}_{\text{e}_{\text{m}}}^{\text{S}}}\right)}$$

Or, in general, for z glycan substrates:

$$\text{r}_{\text{p}}\text{ =}\frac{\text{r}_{\text{e}_{\text{m}}}^{\text{max}}\text{c}_{\text{g}_{\text{i}}}^{\text{n}}\text{c}_{\text{s}_{\text{j}}}^{\text{n}}}{\text{K}_{\text{e}_{\text{m}}}^{\text{S}}\text{K}_{\text{e}_{\text{m}}}^{\text{G}}\left(\text{1+}\sum_{\text{k=1}}^{\text{z}} \frac{\text{c}_{\text{g}_{\text{k}}}^{\text{n}}}{\text{K}_{\text{e}_{\text{m}}}^{\text{G}}}\text{+}\frac{\text{c}_{\text{s}_{\text{j}}}^{\text{n}}}{\text{K}_{\text{e}_{\text{m}}}^{\text{S}}}\text{+}\sum_{\text{k=1}}^{\text{z}} \frac{\text{c}_{\text{g}_{\text{k}}}^{\text{n}}\text{c}_{\text{s}_{\text{j}}}^{\text{n}}}{\text{K}_{\text{e}_{\text{m}}}^{\text{G}}\text{K}_{\text{e}_{\text{m}}}^{\text{S}}}\right)}$$

$$\text{S1.8}$$
