# Supplementary material for: Systems Analysis of N-Glycan Processing in Mammalian Cells
Source: PLoS One. 2007 Aug 8;2(8):e713. doi: 10.1371/journal.pone.0000713 (PMC1933599; doi:10.1371/journal.pone.0000713)
Supplement: Text S2 — Enzyme concentration determination. (0.05 MB DOC) [file pone.0000713.s003.doc]

**Text S2-Enzyme Concentration Determination**

The concentration of each enzyme in the Golgi was determined from their literature reported values of the ratio of enzyme mass purified from an initial total protein mass from cell lysates ($\text{PR}_{\text{e}_{\text{m}}}$). This value was then normalized to the fixed Golgi compartmental volume on a per cell basis assuming 10 Golgi per cell, using the below equation:

$$\text{ c}_{\text{e}_{\text{m}}}\text{= PR}_{\text{e}_{\text{m}}}\text{× TCP }\div\text{ 10 }\div\text{ V }\div\text{ MW}_{\text{e}_{\text{m}}}$$

$$\text{S2.1}$$
