# Supplementary material for: Systems Analysis of N-Glycan Processing in Mammalian Cells
Source: PLoS One. 2007 Aug 8;2(8):e713. doi: 10.1371/journal.pone.0000713 (PMC1933599; doi:10.1371/journal.pone.0000713)
Supplement: Text S3 — Maximum reaction rate determination. (0.05 MB DOC) [file pone.0000713.s004.doc]

**Text S3-Maximum Reaction Rate Determination**

The maximum reaction rates for each enzyme were determined from their literature reported maximum specific activity values (i.e., $\text{r}_{\text{e}_{\text{m}}}^{\text{max}}$per enzyme mass) and scaled to their literature derived enzyme concentrations using the below equation:

$$\text{ r}_{\text{e}_{\text{m}}}^{\text{max}}\text{= }\tilde{\text{r}}_{\text{e}_{\text{m}}}^{\text{max}}\text{× PR}_{\text{e}_{\text{m}}}\text{× TCP ÷ 10 ÷ V }$$

$$\text{S3.1}$$
